# Right Ventricular Compression From Pectus Excavatum: A Reversible Cause of Ventricular Dysfunction

**DOI:** 10.7759/cureus.106639

**Published:** 2026-04-08

**Authors:** Kevin Rivera, Peter Unkovic

**Affiliations:** 1 Internal Medicine, Mount Carmel Health System, Columbus, USA; 2 Cardiology, Mount Carmel Health System, Columbus, USA

**Keywords:** cardiology, cardiomyopathy, ct cardiac, pectus excavatum, right ventricular compression

## Abstract

Pectus excavatum can rarely result in clinically significant right ventricular (RV) compression, leading to impaired diastolic filling, reduced stroke volume, and secondary biventricular dysfunction involving both the right and left ventricles. We report a 19-year-old male who presented with progressive exertional dyspnea and was found to have severe pectus excavatum with a Haller index of 5.8 on cardiac magnetic resonance imaging and 3.6 on computed tomography. Multimodal cardiac evaluation demonstrated focal RV compression, moderate RV systolic dysfunction on cardiac MRI with a right ventricular ejection fraction (RVEF) of 31%, mild left ventricular (LV) systolic dysfunction with an ejection fraction of 44%-45%, and trivial mitral and tricuspid regurgitation. Spirometry was overall normal, without obstructive changes or exercise-associated bronchoconstriction, and electrocardiography revealed marked right-axis deviation. Coronary computed tomography angiography demonstrated normal coronary anatomy, while cardiac MRI showed no delayed gadolinium enhancement to suggest myocardial scar or infiltrative cardiomyopathy. In the absence of alternative clinical or imaging evidence for another cardiomyopathic process, the patient met morphologic and physiologic criteria for surgical repair and underwent minimally invasive correction with pectus bar placement without complication. At five-month follow-up, he reported resolution of dyspnea, although repeat postoperative cardiac imaging had not yet been performed. This case emphasizes the importance of recognizing extrinsic RV compression from pectus excavatum as a surgically correctable and likely reversible contributor to ventricular dysfunction and highlights the role of comprehensive imaging in guiding timely surgical intervention.

## Introduction

Pectus excavatum (PEx) is the most common congenital chest wall deformity, characterized by posterior displacement of the sternum and costal cartilages, resulting in varying degrees of thoracic cavity compression [1]. While it occurs in approximately 1 in 300-400 live births, the degree of structural and physiologic impact varies considerably, and in many cases, PEx is detected incidentally [1,2]. In severe forms, the inward depression of the sternum can lead to clinically significant compression of mediastinal structures, particularly the right ventricle (RV), given its anterior location and thin-walled morphology [2,3].

RV compression in PEx may produce a spectrum of cardiovascular consequences, including impaired diastolic filling, reduced stroke volume, and interventricular dependence, where elevated right ventricular diastolic pressures and septal shift mechanically impair left ventricular filling and contractility, leading to secondary left ventricular (LV) dysfunction [3-5]. These geometric alterations may also distort atrioventricular valve annuli, contributing to regurgitant lesions, most often involving the tricuspid valve and less frequently the mitral valve [3,6,7]. In contemporary imaging series of patients with severe deformities, objective right ventricular dysfunction or measurable compression has been reported in a substantial subset of patients, particularly when the Haller index exceeds 3.2 [3,5]. In addition, conduction abnormalities, axis deviation, and arrhythmias have been reported in association with RV deformation [8-10]. In some cases, the hemodynamic and electrical disturbances from severe PEx can mimic intrinsic cardiomyopathies (e.g., arrhythmogenic or constrictive heart disease), complicating the diagnostic evaluation if the chest wall deformity is not recognized. Such abnormalities may resolve following surgical decompression, highlighting the potential reversibility of functional impairment [4,9,11].

The pathophysiologic impact of PEx on cardiac performance is influenced by multiple factors. This includes the severity of the deformity, which is quantified by indices such as the Haller index or correction index [1,5,12], the degree of sternal rotation [13], and patient-specific cardiopulmonary reserve. The normal Haller index in unaffected individuals is approximately 2.0-2.5, with values >3.2 generally considered severe and potentially physiologically significant [1,5,12]. Severe deformities (Haller index >3.2) have been associated with measurable declines in aerobic and functional exercise capacity, reduced inspiratory capacity, and restrictive ventilatory defects [12,14,15]. Restrictive lung mechanics may increase intrathoracic pressure during exertion, reduce effective venous return, and augment pulmonary vascular resistance, thereby compounding right ventricular preload limitation and systolic impairment in severe deformities. Three-dimensional surface imaging of the anterior chest wall, strain-based echocardiographic techniques, and cardiac MRI provide complementary information to enable structural quantification and functional assessment [5,14,16].

Surgical correction, most commonly via a minimally invasive repair (Nuss procedure), can relieve RV compression and restore near-normal cardiac geometry, often with concomitant improvement in biventricular function and valvular competence [4,6,17]. However, the cardiology literature contains relatively few detailed reports of RV compression as a primary driver of ventricular dysfunction in PEx, particularly when documented across multiple imaging modalities. We present a case of a young adult with severe PEx complicated by RV compression, mild biventricular dysfunction, valvular regurgitation, and electrocardiographic changes, with findings integrated from echocardiography, cardiac MRI, and cardiac computed tomography angiography (CTA). This case highlights the role of cardiology-led multimodal imaging in diagnosis and surgical referral, as well as the pathophysiologic mechanisms linking chest wall deformity to potentially reversible cardiomyopathy.

## Case presentation

A 19-year-old male with a known diagnosis of pectus excavatum was referred for preoperative cardiovascular evaluation in anticipation of surgical repair. He first noted a chest wall deformity approximately seven years prior to presentation, which had been initially asymptomatic. Over the preceding two years, he developed progressive exertional dyspnea that limited his participation in sports and daily activities. He reported a reduction in exercise tolerance from competitive athletic activity to experiencing dyspnea after moderate exertion, consistent with an estimated functional capacity of approximately six to seven metabolic equivalents based on history. He denied chest pain, palpitations, syncope, orthopnea, paroxysmal nocturnal dyspnea, or peripheral edema. His past medical and surgical history was otherwise unremarkable, and he had no known allergies. He denied preceding viral illness, alcohol use, stimulant or illicit drug use, and use of supplements or anabolic steroids. Family history was notable for a maternal grandmother with congenital heart disease of unknown type who reportedly died at age 30 while awaiting a heart transplant. There was no known family history of sudden cardiac death or a defined inherited cardiomyopathy in first-degree relatives.

On physical examination, the patient was afebrile with normal vital signs (blood pressure 125/71 mmHg, heart rate 68 bpm, respirations 16/min, oxygen saturation or SpO₂ at 99% on room air). His BMI was 19.4 kg/m². Cardiopulmonary examination revealed a localized indentation of the lower sternum, with the point of maximal depression approximately 2 cm below the xiphoid process. Heart sounds were normal, without murmurs, rubs, or gallops. There was no fixed splitting of S2, no parasternal heave, and no right ventricular lift appreciated on palpation. Lungs were clear to auscultation bilaterally. There were no signs of jugular venous distension, hepatomegaly, or peripheral edema.

**Figure 1:**
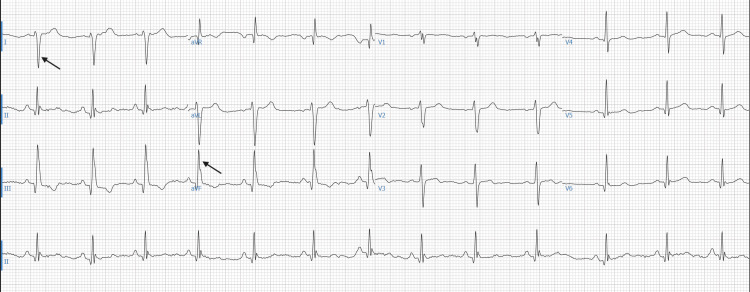
Twelve-lead electrocardiogram demonstrating sinus rhythm with marked right-axis deviation. Arrows highlight the predominantly negative QRS complex in lead I and the positive QRS complex in lead augmented vector foot (aVF), consistent with right-axis deviation. PR interval was 146 ms, QRS duration 108 ms, and corrected QT interval (QTc) 426 ms.

Laboratory evaluation demonstrated normal complete blood count, renal function, electrolytes, coagulation profile, troponin, and thyroid-stimulating hormone. Folate, vitamin B12, and homocysteine were also normal. Outside spirometry was interpreted as overall normal, with no evidence of exercise-associated bronchoconstriction or obstructive ventilatory defect, with forced expiratory volume in one second (FEV1) 4.40 L (91% predicted) and forced vital capacity (FVC) 5.06 L (89% predicted). Formal cardiopulmonary exercise testing was considered but deferred, given the already established structural abnormality and planned surgical intervention. Electrocardiogram (ECG) showed sinus rhythm with marked right-axis deviation, consistent with altered cardiac position and right ventricular compression from the chest wall deformity (Figure 1).

**Figure 2:**
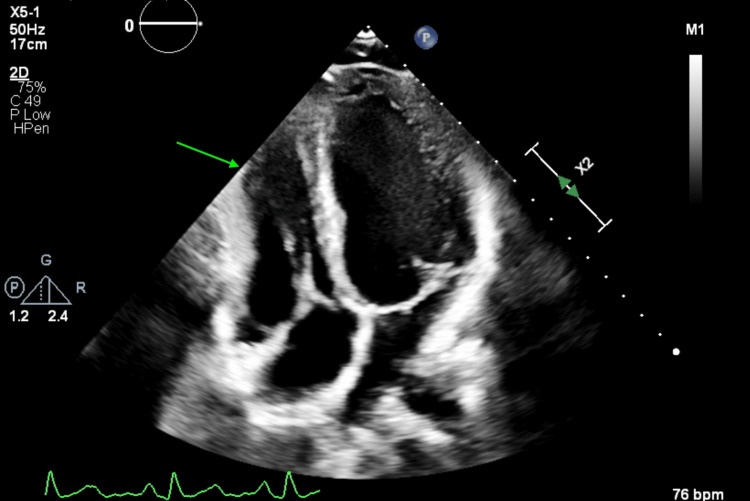
Apical four-chamber transthoracic echocardiographic view demonstrating distortion and focal compression of the right ventricular free wall. Quantitative measurements include right ventricular S’ velocity of 12 cm/s and right ventricular diastolic basal dimension of 4.2 cm. Left ventricular biplane with an ejection fraction of 47%. Arrow highlights the region of altered right ventricular geometry consistent with external compression from severe pectus excavatum.

Transthoracic echocardiography demonstrated normal chamber sizes, abnormal septal motion, trivial mitral and tricuspid regurgitation, mildly reduced left ventricular systolic function with an ejection fraction of 40%-45%, and reduced LV global longitudinal strain of -11.8%. The RV appeared normal in size, with RV diastolic length 8.2 cm, RV diastolic basal dimension 4.2 cm, RV diastolic mid dimension 3.0 cm, and RV S' velocity of 12 cm/s (Figure 2).

**Figure 3:**
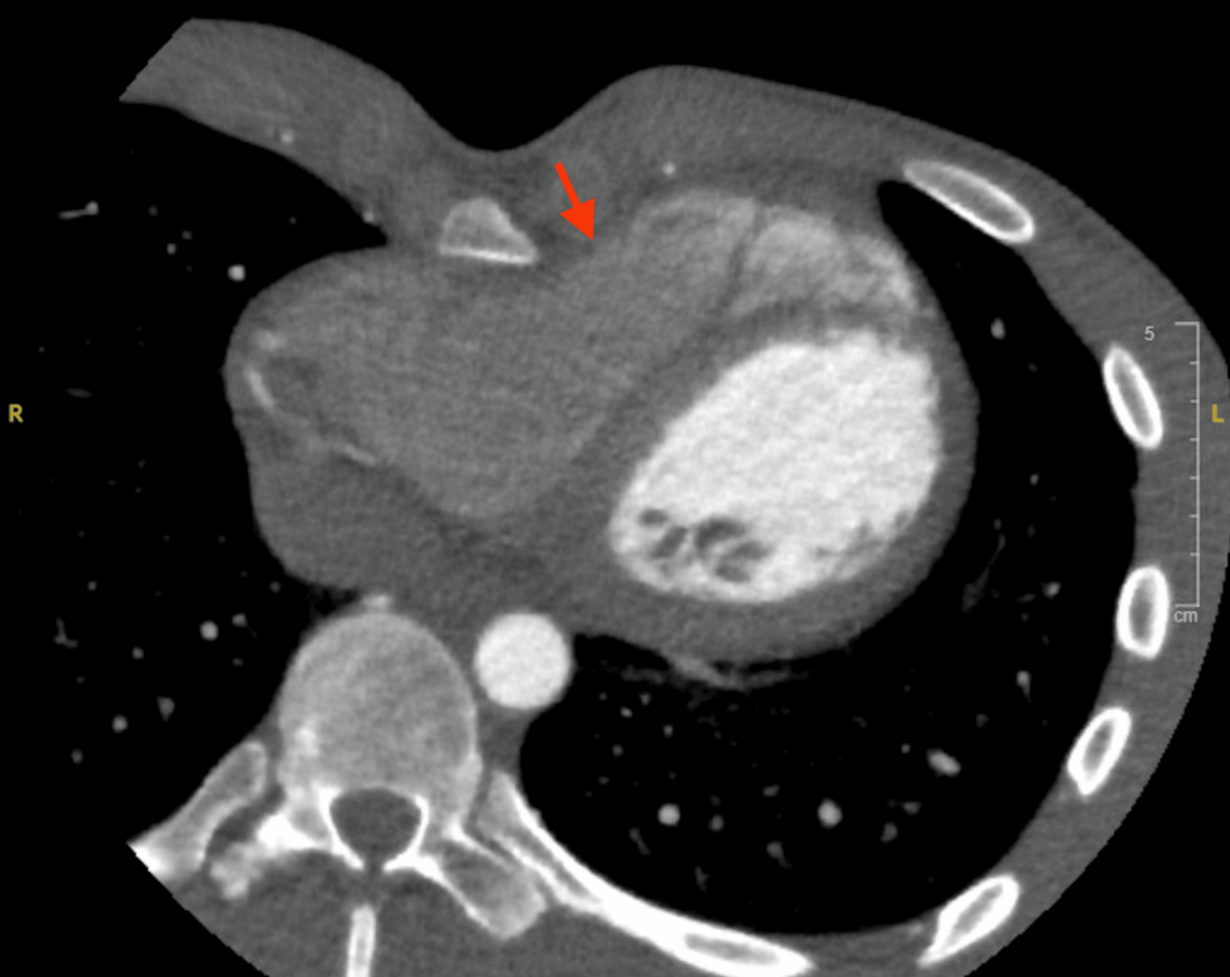
Axial chest CT demonstrating severe pectus excavatum with an arrow highlighting focal right ventricular compression.

Cardiac MRI demonstrated a Haller index of 5.8, confirming a severe chest wall deformity. The sternum was displaced posteriorly, causing partial compression of the RV free wall. RV end-diastolic volume was 216 mL, RV end-systolic volume was 150 mL, RV stroke volume was 66 mL, RV cardiac output was 4.7 L/min, and right ventricular ejection fraction (RVEF) was 31%, consistent with mild to moderate RV systolic dysfunction. The LV was normal in size with mild systolic dysfunction and an ejection fraction of 44%. Both atria were normal in size. There was no delayed gadolinium enhancement to suggest myocardial scar or infiltrative cardiomyopathy. Cardiac valve assessment showed no significant valvular stenosis or regurgitation. CT chest again demonstrated severe pectus excavatum with persistent focal RV compression as visualized in Figure 3, with a Haller index of 3.6.

Coronary computed tomography angiography showed normal coronary anatomy, a calcium score of zero, and no anomalous coronary origin or atherosclerotic disease. Taken together, the absence of ischemic coronary disease, the lack of delayed enhancement on cardiac MRI, the absence of significant congenital or valvular abnormalities on multimodality imaging, and the lack of clinical features suggesting myocarditis or toxic cardiomyopathy supported pectus-related mechanical compression as the leading explanation for ventricular dysfunction. The difference between MRI- and CT-derived Haller indices was interpreted as interstudy measurement variability related to differing imaging modalities, acquisition conditions, and slice selection rather than a meaningful change in deformity severity; importantly, both values remained above the conventional threshold for severe deformity [1,5,12].

The patient subsequently underwent a minimally invasive Nuss procedure with placement of two parallel 13-inch pectus bars and bilateral bridging bars, supplemented by four stabilizing flare busters. Bilateral intercostal nerve cryoablation from T3-T9 was performed for postoperative analgesia. Intraoperative findings confirmed a banana-shaped sternum with maximal depression at the xiphoid. The procedure was completed without complications and minimal blood loss. Postoperative chest radiography confirmed correct bar positioning, with no pneumothorax or pleural effusion. The patient recovered well, was ambulating on postoperative day one, and was discharged home in stable condition. At five-month postoperative follow-up, physical examination showed a level sternum without abnormal protrusion or residual indentation, well-healed incisions, and complete resolution of costal flaring. The patient denied shortness of breath or chest pain. Breath sounds were clear and equal, heart tones were normal, and chest X-ray showed clear lung fields with stable position of the pectus bar and bridging bars.

## Discussion

Severe pectus excavatum can exert direct mechanical effects on the right heart because the RV occupies the most anterior position within the chest and has a relatively thin free wall, making it particularly susceptible to extrinsic compression [1-3]. In this patient, multimodal imaging demonstrated focal RV free-wall compression. Transthoracic echocardiography showed abnormal septal motion and preserved longitudinal RV tissue velocity, whereas cardiac MRI, which offers more robust RV volumetric assessment, quantified an RVEF of 31% with normal RV size and elevated RV end-systolic volume, supporting mild to moderate RV systolic dysfunction [2,14,16]. The accompanying mild LV systolic dysfunction may reflect ventricular interdependence, with altered RV geometry and septal mechanics contributing to impaired LV filling and performance [3-5].

The severity of the deformity is commonly quantified by the Haller index, defined as the ratio of transverse chest diameter to anteroposterior diameter, with values greater than 3.2 generally considered severe [1,5,12]. In our patient, the Haller index measured 5.8 on cardiac MRI and 3.6 on CT. Rather than implying a true anatomic change, we interpret this difference as intermodality measurement variability related to differences in acquisition technique, respiratory phase, and slice selection. Importantly, both measurements remained within the severe range and were concordant with direct visualization of focal RV compression [1,5,12]. Beyond simple compression, sternal rotation and focal asymmetry may further influence the direction and severity of cardiac displacement [13].

The most direct hemodynamic consequence of RV compression is reduced diastolic filling, which decreases preload and stroke volume [2,3,6]. This reduction in preload may be unmasked during exercise, when increased venous return exaggerates the constraint imposed by the rigid anterior chest wall. Intraoperative transesophageal echocardiography studies have documented immediate increases in RV end-diastolic dimensions and cardiac output following surgical decompression [4,6,17]. Furthermore, distortion of the tricuspid annulus from altered RV geometry can produce functional tricuspid regurgitation, as seen in our case, and may also affect the mitral annulus through interventricular septal tethering [3,6,7].

Cardiac displacement and RV compression can also influence the cardiac conduction system. Reported electrocardiographic abnormalities include right-axis deviation, bundle branch block, and, in rare cases, ventricular arrhythmias [8-10]. The marked right-axis deviation observed in our patient is consistent with prior series associating such electrical changes with mechanical displacement of the heart within the thorax [8,9]. While many of these conduction abnormalities are benign, their resolution post-repair supports a mechanical origin [9,10].

Evaluation of suspected cardiac compression in PEx requires a multimodal strategy. Transthoracic echocardiography provides real-time assessment of chamber sizes, valvular competence, and ventricular function, though the anterior location of the RV can limit acoustic windows and potentially underestimate the degree of compression [2,14,16]. Cardiac MRI offers superior characterization of RV morphology, quantification of ventricular volumes and ejection fraction, and myocardial tissue characterization to exclude alternative causes of cardiomyopathy [5,14,16]. CT allows accurate calculation of the Haller index, assessment of sternal rotation, and direct visualization of the spatial relationship between the sternum and the heart [1,5]. In this case, our cardiology-led workup integrated all three imaging modalities, in addition to coronary CTA to exclude concomitant coronary pathology, providing a comprehensive evaluation of the chest wall deformity’s impact on cardiac structure and function.

The minimally invasive Nuss procedure is the preferred approach for most patients requiring correction of pectus excavatum, and prior series have documented improvements in RV filling, stroke volume, exercise tolerance, and, in some patients, ventricular performance after repair [4,6,14,17]. In the present case, formal postoperative cardiac imaging had not been performed at the time of manuscript revision. However, at five-month follow-up, the patient reported resolution of shortness of breath and chest pain, examination showed a level sternum with resolved costal flaring, and chest radiography confirmed stable bar position with clear lung fields. Accordingly, this case supports pectus excavatum as a surgically correctable and likely reversible contributor to ventricular dysfunction, while acknowledging that direct post-repair quantification of ventricular recovery in this individual is not yet available.

Cardiologists play a pivotal role in identifying those patients whose deformity is causing hemodynamically significant cardiac compression. This requires awareness of the pathophysiologic relationship between chest wall deformity and cardiac performance, targeted use of advanced cardiac imaging, and close multidisciplinary collaboration with thoracic surgery. Our case illustrates the value of a comprehensive cardiology-led evaluation in guiding surgical referral and in identifying a potentially remediable mechanical cause of ventricular dysfunction.

Beyond the structural and hemodynamic considerations illustrated in this case, it is important to recognize that cardiovascular risk and outcomes are shaped by broader socioeconomic and racial disparities. Contemporary epidemiologic data demonstrate persistent differences in hypertension, obesity, diabetes, and cardiovascular mortality across racial and ethnic groups, with disproportionately higher burdens in non-Hispanic Black and Hispanic populations. Although hypercholesterolemia prevalence appears relatively similar across groups, rates of hypertension, diabetes, obesity, and smoking differ substantially, and these disparities translate into sustained gaps in cardiovascular mortality despite overall improvements over time. Socioeconomic determinants, including educational attainment, income level, employment status, neighborhood environment, food insecurity, housing instability, insurance coverage, and access to mental health services, further modulate cardiovascular risk and access to specialty evaluation. In the context of pectus excavatum, where recognition of physiologically significant right ventricular compression often requires advanced imaging and multidisciplinary referral, inequities in healthcare access may delay diagnosis or intervention. Awareness of these structural determinants is therefore essential to ensure that potentially reversible causes of cardiac dysfunction are identified and addressed equitably across diverse populations [18].

## Conclusions

Severe pectus excavatum can produce clinically significant RV compression with associated ventricular dysfunction and electrocardiographic abnormalities. Recognition of deformity-related cardiac compromise requires careful multimodal imaging, particularly when echocardiographic assessment of the RV is limited. In this patient, cardiac MRI provided the clearest quantification of RV dysfunction and helped exclude competing structural or infiltrative etiologies. Surgical repair was followed by clear clinical improvement at five months, although repeat postoperative cardiac imaging was not obtained. This case, therefore, highlights pectus excavatum as a surgically correctable and likely reversible cause of ventricular dysfunction and underscores the value of timely multidisciplinary evaluation.
